# A new species of Centaurea sect. Pseudoseridia (Asteraceae) from north-eastern Turkey

**DOI:** 10.3897/phytokeys.53.5250

**Published:** 2015-07-21

**Authors:** İbrahim Sırrı Yüzbaşıoğlu, Mehmet Bona, İlker Genç

**Affiliations:** 1 Department of Pharmaceutical Botany, Faculty of Pharmacy, İstanbul University, 34116, İstanbul, Turkey; 2 Department of Botany, Science Faculty, İstanbul University, 34134, İstanbul, Turkey

**Keywords:** *Centaurea*, new species, taxonomy, Turkey

## Abstract

*Centaurea ziganensis* Yüzb., M. Bona & İ. Genç, a new species is described and illustrated from Gümüşhane province, NE Turkey. The new species grows in rocky places on the south face of Zigana Mountains, and is closely related to *Centaurea drabifolioides*, from which it differs mainly in stem, achene and phyllary appendage characters. Micromorphological structures of achenes and karyological features of *Centaurea ziganensis* and *Centaurea drabifolioides* were examined in this study.

## Introduction

*Centaurea* L. *s.l.* is one of the largest and taxonomically most difficult genera of the Asteraceae (Dostál 1976). Recent approaches have split this taxon into four genera: *Centaurea*, *Rhaponticoides* Vaill., *Psephellus* Cass. and *Cyanus* Mill. (Wagenitz and Hellwig 2000, Greuter 2003, Hellwig 2004). The genus *Centaurea* was previously revised by Wagenitz (1975) for the *Flora of Turkey and the East Aegean Islands* without considering the splitting mentioned above. Even excluding the species now placed in these genera, Turkey is among the richest countries in *Centaurea* diversity (Wagenitz 1975, Davis et al. 1988, Güner 2000).

Recently *Centaurea* *s.l.* was revised for *Türkiye Bitkileri Listesi* by Dural (2012), Ertuğrul (2012) and Uysal (2012a, 2012b). According to revised system, the number of known *Centaurea* species in Turkey is 162 [(excluding 56 species which are now treated within *Psephellus* (33), *Cyanus* (16) and *Rhaponticoides* (7)].

In the *Flora of Turkey and the East Aegean Islands* (Wagenitz 1975), 34 sections of *Centaurea* were presented. Some sections of the Turkish Centaurea have been revised, but others, such as sect. Pseudoseridia Wagenitz, have not been revised recently. According to Wagenitz (1975) there were seven Centaurea species in section Pseudoseridia. Since then, six new taxa have been described for the section (Uzunhisarcıklı et al. 2005, 2007, Uysal et al. 2007, Aksoy et al. 2008, 2010, Bona 2015).

The present study is focused on the morphological, micromorphological and karyological criteria for distinguishing a new species in Centaurea sect. Pseudoseridia. Investigations on living and herbarium specimens suggest that this new species is morphologically most similar to *Centaurea drabifolioides*.

## Material and methods

Flowering and fruiting specimens of the *Centaurea ziganensis* and of related species, *Centaurea drabifolioides* were collected by the first author several times in 2013 and 2014 from type localities. The *Centaurea* material was examined and compared with material of similar taxa (sect. *Pseudoseridia*) in ISTE, ISTO, GAZI, ANK, HUB, E, K and G. The specimens were also cross-checked with various accounts of *Centaurea* in relevant floras, i.e. *Flora Orientalis* (Boissier 1875), *Flora Europaea* (Dostál 1976), *Nouvelle Flore du Liban et de la Syrie* (Mouterde 1983) and a taxonomic study on *Centaurea* in Iran (Negaresh et al. 2014). The measurements, colors and other details given in the description are based on both herbarium and living materials. Herbarium specimens were deposited in the herbaria of ISTE. Photographs of living material were taken with a Canon D60 digital camera (Canon EF 100 mm macro-lens) and the illustrations of the new species were made by using Adobe Photoshop CS4. The morphology of the new species was examined with the aid of a Leica S8AP0 stereo–binocular microscope.

During Scanning Electron Microscopy, 2 mature achenes from *Centaurea ziganensis* (ISTE 104470) and *Centaurea drabifolioides* (ISTE 104472) were selected and mounted onto stubs with double-sided adhesive tape, and were then coated with gold. The achene surfaces were examined from the lateral sides. For each sample, photographs of the testa were taken using the JEOL JSM-5600 at a magnification 500×, 1000×, and 3000×. The terminology of achene characteristics in this work was based on the descriptions used by Stearn (1992), Barthlott (1981), and Koul et al. (2000).

Chromosome number and karyological features of the *Centaurea ziganensis* and *Centaurea drabifolioides*, were determined from plant material collected from type localities. All karyological observations were carried out on root tips. Root-tip meristems were provided from achenes by germinating them on wet filter paper in petri dishes at room temperature. Firstly, root tips pretreated for 24 h in a-monobromonaphthalene at 4 °C, fixed in 3:1 absolute alcohol-glacial acetic acid, then the root tips were hydrolyzed with 1 N HCL for 12 min at 60 °C and stained in Feulgen solution and squashed in aceto-orcein (Altınordu et al. 2014).

For karyotype analysis, the photographs were taken using OLYMPUS BX53 microscope with camera Kameram12 CCD attachment. Chromosome counts in mitosis metaphase and karyotype analyses were obtained based on three root tips, five metaphase cells for each individual. Measurements of somatic chromosomes were made with the program CAMERAM, they were calculated with formula of the relative variation in chromosome length (CV_CL_) (Paszko 2006) and mean centromeric asymmetry (M_CA_) according to Peruzzi and Eroğlu (2013). Chromosomes were classified according to the nomenclature of Levan et al. (1964) and Stebbins asymmetry types are given (Stebbins 1971).

## Taxonomic treatment

### Centaurea ziganensis

Taxon classificationPlantaeAsteralesAsteraceae

Yüzb., M. Bona & İ. Genç sp. nov.

urn:lsid:ipni.org:names:77148383-1

Fig. 1

#### Diagnosis.

*Centaurea ziganensis* is related to *Centaurea drabifolioides*, from which it differs mainly in its 2–4 (–8) branched and non-winged stem (not simple and winged), median phyllary appendages with 4–6 pairs of cilia (not 5–10); achenes 4.5–5 mm long, oblong, straw-colored, striate (not 5.5–6 mm long, lanceolate, blackish-chestnut, shiny); pappus straw-colored (not blackish-chestnut).

#### Type.

**TURKEY. Gümüşhane**: Zigana pass–Gümüşhane road, c. 5. km, rocky places, 1450 m a.s.l., 20 Aug 2014, *S. Yüzbaşıoğlu 4117* (holotype: ISTE 104470, isotype: ANK).

#### Description.

Perennial herb with sterile leaf-rosettes and woody rootstock. Stems erect or erect-ascending, pubescent with short simple hairs, 30–70 cm long, not winged, usually 2–4 (–8) well developed one-headed branched from near middle. Basal leaves scabrid with multiseriate septate hairs, and densely covered transparent sessile glands on both surfaces, narrowly lanceolate, petiole 3.5–6.5 cm long, margins entire or sparsely toothed, c. 10 teeth on each side (c. 1 mm long), acute, attenuate, 10–20 × 0.5–1.5 cm (inc. petiole). Median and upper cauline leaves scabrid, linear-lanceolate, mucronate (c. 1 mm) at apex, margins entire; the median ones 5–10.5 × 0.8–1 cm, distinctly decurrent; the upper ones 1.2–3.5 × 0.2–0.4 cm, sessile, not decurrent, leaves decreasing to capitula, uppermost bract-like. Capitula 2–4 (–8), solitary at end of well-developed branches. Involucre ovoid, 15–20 × 12–15 (–18) mm. Phyllaries green, tomentose at apex; the outer ones ovate, 5–6 × 4–5.2 mm; the median ones lanceolate, 9.5–10 × 4.6–5 mm; innermost linear-lanceolate, 16–17 × 3.2–4 mm. Appendages small, not concealing basal part of phyllaries, 1.5–2 mm broad, dark brown, triangular, not decurrent, spreading or reflexed, with 4–6 pairs of cilia (1–3 mm), ending with a 2–4 mm spinule. Florets yellow, marginal not radiant; corolla tube glabrous, 20 mm long, lobes 6–7 × 0.5 mm, linear, with 5 brown stripes along corolla tubes; anthers 10 mm, filaments 4 mm long. Achenes oblong, straw-coloured, glabrous, distinctly striate, 4.5–5 × 2–2.2 mm; pappus straw-coloured, biseriate, scabrous, outer series 5.3–6.2 mm long, inner series 1.5–1.8 mm long.

**Figure 1.**
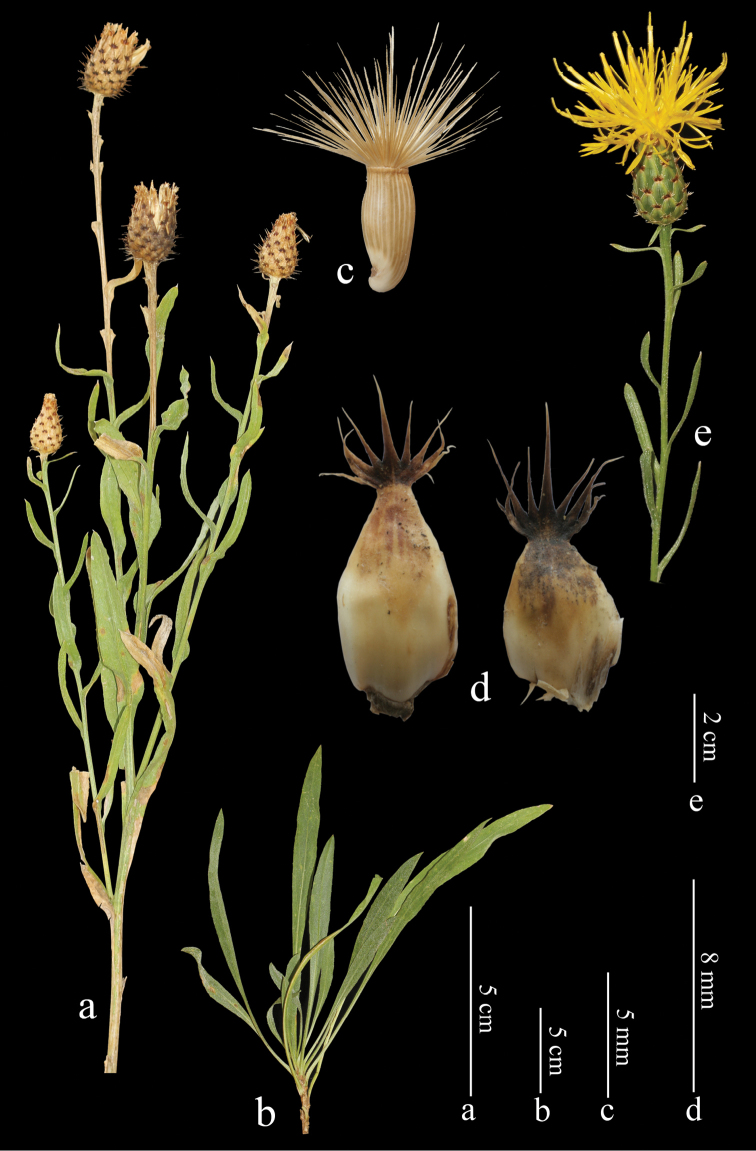
*Centaurea ziganensis* sp. nov. **a** habit **b** roset leaves **c** achene **d** median phyllaries **e** capitulum.

#### Phenology.

*Centaurea ziganensis* flowers from the end of June to–July, and mature fruits are produced in August−early September.

#### Etymology.

Named after the Zigana Mountains where it was discovered.

#### Ecology.

The new species was only found on the southern slopes of Zigana Mountains and occurs on rocky slopes at c. 1500 m elevation. Within this area, the new taxon is associated with plants such as: *Allium rupestre* Steven, *Alyssoides utriculata* (L.) Medik., *Arenaria serpyllifolia* L., *Asplenium septentrionale* (L.) Hoffm., *Asyneuma lobelioides* (Willd) Hand.-Mazz., *Berberis vulgaris* L., *Campanula betulifolia* C. Koch, Coronilla orientalis Mill. var. orientalis, *Euphorbia condylocarpa* M. Bieb., *Fibigia clypeata* (L.) Medik., *Haplophyllum armenum* Spach, *Helianthemun nummularium* (L.) Mill., *Hypericum pruinatum* Boiss. & Bal., Hyssopus officinalis L. subsp. officinalis, Juniperus oxycedrus L. subsp. oxycedrus, *Lamium galactophyllum* Boiss. & Reuter, *Laser trilobum* (L.) Borkh., *Ornithogalum narbonense* L, *Psephellus pyrrhoblepharus* (Boiss.) Wagenitz, *Reichardia glauca* Matthews, *Rosa canina* L., *Salvia tomentosa* Mill., *Salvia verticillata* L., *Saxifraga paniculata* Mill., *Sempervivum transcaucasicum* Muirhead, Silene dichotoma Ehrh. subsp. sibthorpiana (Reichb.) Rech., *Sobolewskia clavata* Fenzl, *Teucrium polium* L. and species of *Acer* and *Quercus*.

#### Distribution and proposed conservation status.

As presently known, *Centaurea ziganensis* is a narrow endemic and known only from the type locality, north eastern Anatolia (Gümüşhane): where the extent of occurrence is less than 100 km^2^ (criterion B1), with an estimated area of occupancy of less than 10 km^2^ (criterion B2). According to our field observations, habitat destruction through human encroachment such as road construction is the principal threat in the area. Therefore, on the basis of our knowledge, we argue that the species is potentially Critically Endangered (CR), but more data are needed to estimate its real IUCN category of threat (IUCN 2014).

#### Karyology.

**Figure 2.**
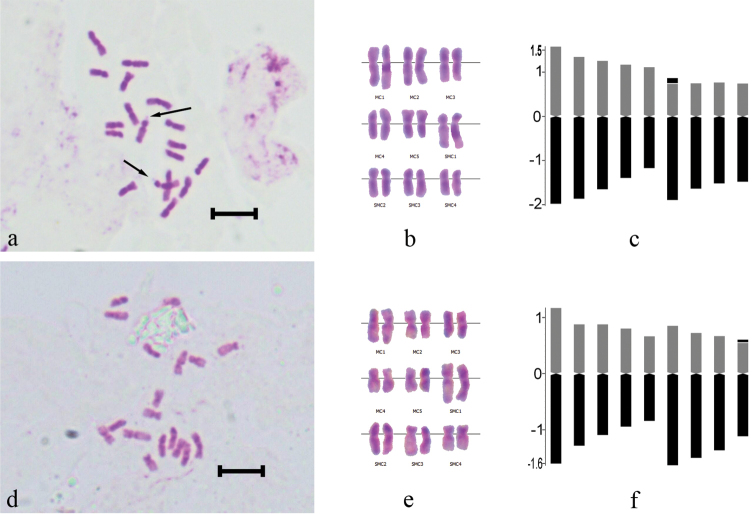
Somatic chromosomes, karyotype and idiogram of *Centaurea ziganensis* (**a–c**); *Centaurea drabifolioides* (**d–f**). Arrow indicates satellite. Scale bars = 5 µm.

The chromosome number of the new taxon is 2n = 18 (Fig. 2a). The shortest chromosome length is 2.22 µm, the longest is 3.55 µm, and the haploid chromosome length is 24.17 µm. The karyotype formula of this taxon consists of 10 median pairs and 8 submedian pairs. Satellites were usually seen on the short arms of the longest sub-metacentric chromosomes. As for karyotype asymmetry, the karyotype of this species is classified according to the symmetry classes of Stebbins as 3A. Intrachromosomal asymmetry (M_CA_) is 20.90 and the interchromosomal asymmetry index (CV_CL_) is 16.32. The karyogram is given in Figure 2b, and ideogram was drawn based on the centromeric index (Fig. 2c).

Our study showed that the chromosome number of *Centaurea drabifolioides* Hub.-Mor. is 2n = 18 (Fig. 2d). The shortest chromosome length is 1.51 µm, the longest is 2.77 µm, and the haploid chromosome length is 18.64 µm. The karyotype formula of this taxon consists of 10 median pairs and 8 submedian pairs. Satellites were usually seen on the short arms of shortest sub-metacentric chromosomes. As for karyotype asymmetry, the karyotype of this species is classified according to the symmetry classes of Stebbins as 3A. Intrachromosomal asymmetry (M_CA_) is 22.71 and the interchromosomal asymmetry index (CV_CL_) is 17.95. The karyogram is given in Figure 2e, and ideogram was drawn based on the centromeric index (Fig. 2f).

#### SEM observations.

**Figure 3.**
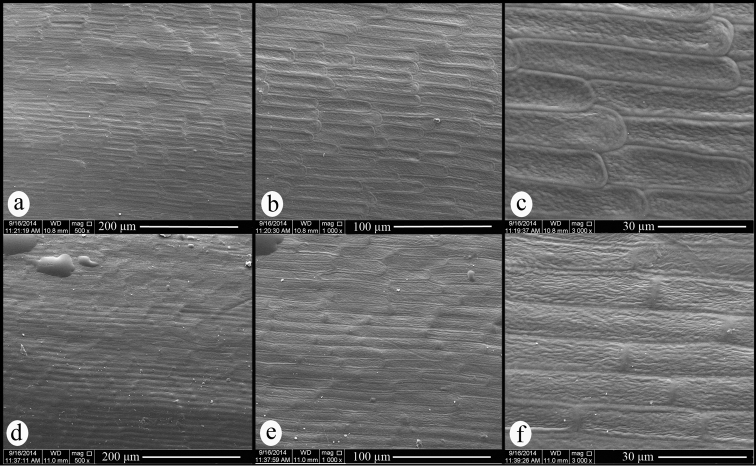
Scanning electron micrographs of achene surface. (**a–c**) *Centaurea ziganensis*. (**d–f**) *Centaurea drabifolioides*.

Seed surface pattern of *Centaurea ziganensis* is ruminate. Testa cells are regularly arranged, elongated parallel with the seed surface and the cells are apparently imbricate. The cell boundaries are thin and have smooth structure, and the boundaries raised above cell centre (Fig. 3a–c). Even seed surface pattern and testa cell arrangement of *Centaurea drabifolioides* are similar to *Centaurea ziganensis*, the cell centres raised above the boundaries and testa cells are not apparently imbricate (Fig. 3d–f).

## Discussion

In section *Pseudoseridia*, *Centaurea hermannii* F.Hermann is unique taxon which has both orange flowers and lyrate leaves. *Centaurea cheirolopha* (Fenzl) Wagenitz, *Centaurea amanosensis* M. Bona, *Centaurea lycopifolia* Boiss. & Kotschy ex Boiss., and *Centaurea stevenii* M. Bieb. have short pappus and lyrate or pinnatifid leaves differing from the remaining taxa in the section. *Centaurea glabroauriculata* Uysal & Demir, *Centaurea kizildaghensis* Uzunh., E. Doğan & H. Duman, and *Centaurea pseudokotschyi* Wagenitz are also easily separated from the other *Pseudoseridia* taxa by their non decurrent leaves. Among the section *Pseudoseridia* taxa, only *Centaurea drabifolioides*, *Centaurea yaltirikii* N. Aksoy, H. Duman & A. Efe, *Centaurea cheirolepidoides* Wagenitz, *Centaurea marashica* Uzunh., Tekşen & E. Doğan have long pappus, and decurrent, simple leaves. The nearly cylindrical involucrum and grey tomentose leaves of *Centaurea cheirolepidoides* and *Centaurea marashica* are different from the ovoid to ovoid-oblong involucrum and scabrous leaves of *Centaurea drabifolioides* and *Centaurea yaltirikii*. Finally, *Centaurea yaltirikii* is different with scabrous-barbellate pappus and a widely broad-winged stem (2−4 mm); while *Centaurea drabifolioides* has scabrous and narrowly winged (0.5−1 mm) stems. In addition, *Centaurea drabifolioides* has linear-lanceolate leaves compared with the broader and lanceolate, oblong, ovate or oblanceolate cauline leaves of *Centaurea yaltirikii*.

**Figure 4.**
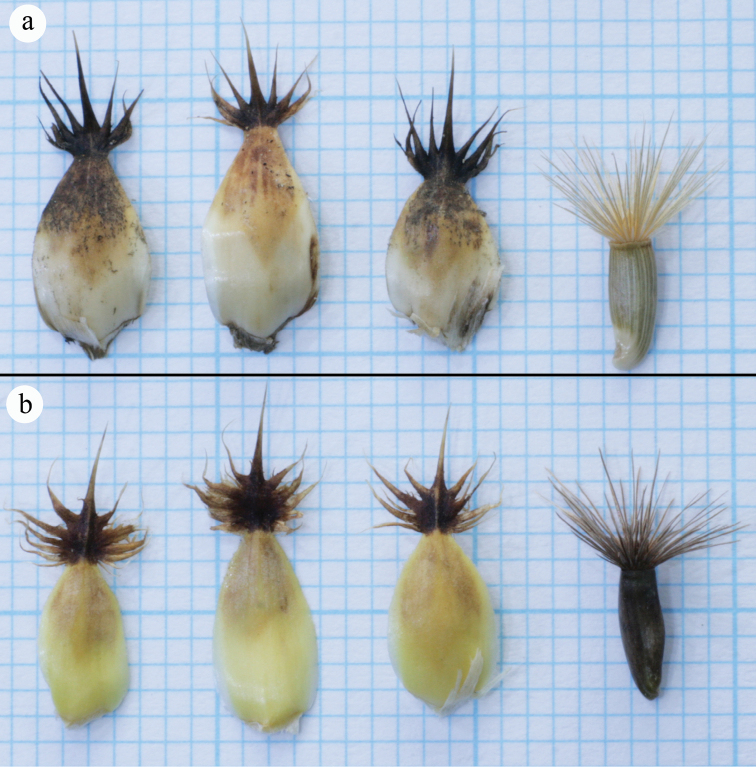
The character comparison of the median phyllary appendages and achenes of *Centaurea ziganensis* (**a**) and *Centaurea drabifolioides* (**b**).

Achene, pappus and phyllary characters provide the most reliable characteristics to separate *Centaurea* taxa from each other at sectional and specific level (Wagenitz 1975, Wagenitz and Helwig 2000, Bancheva and Gorgorov 2010). It is known that achene micromorphology also provides strong support in the delimitation of *Centaurea* taxa (Bagheri Shabestari 2013, Bona 2014). *Centaurea ziganensis* closely related to *Centaurea drabifolioides*. Even though they both have long pappus, simple, scabrous, decurrent, linear-lanceolate leaves, and are distributed close to each other in a similar habitat, *Centaurea ziganensis* differs from *Centaurea drabifolioides* in its stem, achene and pappus colour, appendage of phyllary, achene micromorphology and karyology. These two species are compared in Table 1 and Figures 2, 3, 4.

**Table 1.** Morphological, micromorphological and karyological comparison of *Centaurea ziganensis* and *Centaurea drabifolioides* (Abbreviations; SCL: shortest chromosome length; LCL: longest chromosome length; HCL: haploid chromosome length; M_CA_: intrachromosomal asymmetry; CV_CL_: interchromosomal asymmetry index).

**Character**	***Centaurea ziganensis***	***Centaurea drabifolioides***
Stem	non-winged, 2–4 (–8) well developed branched	winged, simple
Appendage	with 4–6 pairs of digitat cilia, very narrowly triangular	with 5–10 pairs of pinnat cilia, triangular
Achene	4.5–5 mm long, oblong, straw colored, striate	5.5–6 mm long, lanceolate, blackish-chestnut, smooth, shiny
Pappus	straw colored	blackish-chestnut
Seed surface pattern	cell boundaries raised above cell center, testa cells rounded at apex and apparently imbricate	cell center raised above cell boundaries, cells not rounded at apex and not apparently imbricate
Karyology SCl LCL HCL M_CA_ CV_CL_ Satellite	2.22 µm 3.55 µm 24.17 µm 20.90 16.32 usually seen on the short arms of longest sub-metacentric	1.51 µm 2.77 µm 18.64 µm 22.71 17.95 usually seen on the short arms of shortest sub-metacentric

Huber-Morath named *Centaurea drabifolioides* in 1967, and based his description of this species on plants collected from near Şebinkarahisar (Giresun), NE Turkey. It is not a common plant throughout this range occurs in a relatively small area and has not been recorded from different part of Turkey, it is only known from the type locality. According to type description (Huber-Morath 1967) and Flora of Turkey (Wagenitz 1975) pappus color for *Centaurea drabifolioides* was indicated as whitish (Huber-Morath 1967) and cream (Aksoy et al. 2008, Uysal et al. 2007). But, the observations we made in the type locality of *Centaurea drabifolioides* indicate that the pappus color of mature achenes is the same as that of the achene, blackish-chestnut. We think that, the pappus color that is referred to in previous studies was from immature achenes.

In this paper, we describe a further new species for Centaurea section Pseudoseridia. The total number of sect. *Pseudoseridia* taxa known from Turkey with this new species, has increased to fourteen, twelve of these are endemic to Turkey. A new identification key for sect. *Pseudoseridia* in Turkey has been prepared according to Wagenitz (1975), Uzunhisarcıklı et al. (2007) and Bona (2015), and the new species may be inserted as follows:

**Table:** 

1	Pappus short (0.5−3 mm)	**2**
2	Leaves grey-tomentose below	**3**
3	Perennial, stem erect or ascending, basal leaves lyrate or lanceolate	***Centaurea cheirolopha***
3'	Biennial, stem decumbent, basal leaves pinnatifid	***Centaurea amanosensis***
2'	Leaves not grey-tomentose below	**4**
4	Leaves or their terminal segments toothed; appendages of inner phyllaries brown	***Centaurea lycopifolia***
4	Leaves simple or divided, margins entire; appendages of inner phyllaries straw-coloured	***Centaurea stevenii***
1'	Pappus longer (5−15 mm)	**5**
5	Cauline leaves non-decurrent	**6**
6	Stem leaves auriculate; terminal spinule of appendage distinctly longer than the other cilia	***Centaurea glabroauriculata***
6'	Stem leaves non-auriculate; terminal spinule not distinct or slightly so	**7**
7	Stem ascending; leaves semi-amplexicaul; phyllary appendages with five to six pairs of cilia (cilia 3−5 mm) and ending in a 4−6 mm spinule	***Centaurea pseudokotschyi***
7'	Stem erect; leaves sessile; phyllary appendages with two to four pairs of cilia (cilia c. 1 mm) and ending in a 1−1.5 mm spinule	***Centaurea kizildaghensis***
5'	Cauline leaves decurrent	**8**
8	Basal or lower stem leaves lyrate	***Centaurea hermannii***
8'	All leaves undivided, basal sometimes with a pair of teeth or lobes	**9**
9	Leaves scabrous; involucre ovate-oblong	**10**
10	Stem wings 2−4 mm broad; median stem leaves lanceolate-oblong, rarely ovate or oblanceolate, 0.7−2 cm broad, shortly decurrent	***Centaurea yaltirikii***
10'	Stem wings absent or 0.5−1 mm broad; median stem leaves linear-lanceolate to linear, 0.4−0.6 cm broad, distinctly decurrent	**11**
11	Stem 2−4 (−8) well developed branched; phyllary appendages with 4−6 pairs of digitat cilia; achene straw coloured	***Centaurea ziganensis***
11'	Stem usually simple; phyllary appendages with 5−10 pairs of pinnat cilia; achene blackish-chestnut	***Centaurea drabifolioides***
9'	Leaves grey tomentose; involucre nearly cylindrical	**12**
12	Median and upper leaves with 0.5−2 mm long spinule at apex; phyllary cilia 1−1.5 mm, terminal spinule 1−2 mm	***Centaurea cheirolepidoides***
12'	Median and upper leaves with 2.5−6 mm long spinule at apex; phyllary cilia 2−4 mm, terminal spinule 2−5 mm	***Centaurea marashica***

### Additional specimens examined.

***Centaurea ziganensis*: Turkey.** Gümüşhane: Zigana pass–Gümüşhane road, c. 5. km, rocky places, 1450 m a.s.l., 04 Sep 2013, *S. Yüzbaşıoğlu 3903* (ISTE 104468); ibid., 09 Jun 2014, *S. Yüzbaşıoğlu 4002* (ISTE 104469).

***Centaurea drabifolioides***: **Turkey**. A7 Giresun: Şebinkarahisar–Dereli road, c. 9. km, within valley, rocky places, 1340 m a.s.l., 10 Jun 2014, *S. Yüzbaşıoğlu 4045* (ISTE 104471); ibid., 23 Aug 2014, *S. Yüzbaşıoğlu 4118* (ISTE 104472); distr. Şebinkarahisar, Schlucht des Arslanyurdu Deresi 9-11 km nördlich von Şebinkarahisar, 1300–1330 m a.s.l., 01 Jul 1955, *A. Huber-Morath 13243*! (holotype, G); ibid., 8 km N Şebinkarahisar, 1300 m a.s.l., 03 Aug 1989, *M. Nydegger* 44650! (G, HUB); ibid., 9 km N Şebinkarahisar, 1300 m a.s.l., 19 Jul 1992, *M. Nydegger* 46809! (G).

## Supplementary Material

XML Treatment for Centaurea ziganensis
